# *Siphliops* gen. nov., a new genus of Baetidae (Ephemeroptera) from New Guinea

**DOI:** 10.3897/zookeys.1277.185660

**Published:** 2026-04-16

**Authors:** Thomas Kaltenbach, Nikita J. Kluge, Martin Bláha, Jiří Patoka, Jean-Luc Gattolliat

**Affiliations:** 1 Muséum cantonal des Sciences Naturelles, Département de zoologie, Palais de Rumine, Place Riponne 6, CH-1005 Lausanne, Switzerland Department of Ecology and Evolution, University of Lausanne Lausanne Switzerland; 2 Department of Ecology and Evolution, University of Lausanne (UNIL), CH-1015 Lausanne, Switzerland Department of Entomology, Biological Faculty, Saint-Petersburg State University Saint Petersburg Russia; 3 Department of Entomology, Biological Faculty, Saint-Petersburg State University, Universitetskaya nab., 7/9, Saint Petersburg, 199034, Russia Department of Biology, Faculty of Science, Humanities and Education, Technical University of Liberec Liberec Czech Republic; 4 Faculty of Fisheries and Protection of Waters, University of South Bohemia in České Budějovice, Zátiší 728/II, 389 01 Vodňany, Czech Republic Department of Zoology and Fisheries, Faculty of Agrobiology, Food and Natural Resources, Czech University of Life Sciences Prague Prague Czech Republic; 5 Department of Zoology and Fisheries, Faculty of Agrobiology, Food and Natural Resources, Czech University of Life Sciences Prague, Kamýcká 129, 165 00 Prague-Suchdol, Czech Republic Muséum cantonal des Sciences Naturelles, Département de zoologie, Palais de Rumine Lausanne Switzerland; 6 Department of Biology, Faculty of Science, Humanities and Education, Technical University of Liberec, Studentská 1402/2, 461 17 Liberec, Czech Republic Faculty of Fisheries and Protection of Waters, University of South Bohemia in České Budějovice Vodňany Czech Republic

**Keywords:** COI, mayflies, Melanesia, new species, *Siphliops extenso* sp. nov., subimago

## Abstract

Mayflies collected in the central mountain range of New Guinea (Highland Papua Province) revealed a new genus of Baetidae, *Siphliops***gen. nov**. The larvae present a unique combination of characters, particularly: abdominal tergum IX with acute extension of the posterolateral corners; carina-like elevation on the frons; labium with glossae and paraglossae spread, and glossae much shorter than paraglossae. The new genus belongs to the tribe Labiobaetini. A new species, *Siphliops extenso***gen. nov. et sp. nov**. is described and illustrated based on larva and subimago. Remarkably, it is known from a single locality only, despite the large amount of sampling done in New Guinea in the past years. COI sequences were obtained from three specimens. Morphological similarities and the relationship of the new genus to other genera of Baetidae are discussed.

## Introduction

New Guinea, the second largest island after Greenland, lies at the interface between the Australian and the Pacific plate with accompanying ongoing tectonic activity, uplift, volcanism, and rifting. Most of the landmass was formed in the past five million years, after an earlier Cenozoic period characterized by an archipelago structure (Davies 2009). Today, there is an impressive central mountain range (fold belt) across New Guinea, with peaks rising to almost 4900 m. The biogeography of New Guinea is both influenced by a faunal exchange with Australia through repeated land connections during late Cenozoic, and by strong affinities with Southeast Asia despite an oceanic barrier; plants and invertebrates are largely of the latter origin (Allison 2009). An exception is the crayfish fauna, which is of Australian origin (Bláha et al. 2016). There is strong evidence that environmental change in the extremely structured central highlands of New Guinea with its ongoing uplift, formation of rich aquatic resources, remote valleys, and mountain blocks has been the primary driver of diversification of aquatic insects in that area (Toussaint et al. 2013, 2014; Cozzarolo et al. 2019).

New Guinea comparatively exhibits a low diversity of mayflies, with only five of the approximately 40 families and 13 of the approximately 460 genera known worldwide (Jacobus et al. 2019; Kluge 2026). Within Baetidae, which is the most diverse family of Ephemeroptera and accounts for nearly a third of all mayfly species and approximately a quarter of all mayfly genera worldwide (Jacobus et al. 2019; Kluge 2026), only five of the approximately 115 genera have been reported so far from New Guinea (*Centroptella* Braasch & Soldán, 1980; *Cloeon* Leach, 1815; *Labiobaetis* Novikova & Kluge, 1987; *Mystaxiops* McCafferty & Sun, 2005; and *Papuanatula* Lugo-Ortiz & McCafferty, 1999). Remarkably, two of these genera developed a very high diversity. *Labiobaetis* is currently represented by 43 species (Lugo-Ortiz et al. 1999; Kaltenbach and Gattolliat 2018, 2021; Kaltenbach et al. 2021, 2023), and *Papuanatula* by 32 species (Lugo-Ortiz and McCafferty 1999; Kaltenbach et al. 2025a, 2025b). A comparable pattern of very high diversity is observed in the family Leptophlebiidae, within the tribe Thraulini (Cozzarolo et al. 2019; Sartori and Salles 2025).

In this study, we describe and illustrate the larva and the male subimago of a new genus and a new species of Baetidae, *Siphliops extenso* gen. nov. et sp. nov., presently known from one area in New Guinea (Wamena, Highland Papua Province).

The new genus is distinguished from all other genera of Baetidae by important characters on mouthparts, legs, and shape of tergum IX.

The other genera of Baetidae known from New Guinea are widely distributed, except *Mystaxiops*, which is endemic to the island, and *Papuanatula*, which is endemic to Australasia (Gattolliat et al. 2026).

## Materials and methods

The larvae were collected by kick-sampling and preserved in 70–96% ethanol. A male subimago was reared by one of us (NK) from a mature larva in a net cage placed in running water.

Dissection of larvae was done in Cellosolve (2-ethoxyethanol) with subsequent mounting on slides with Euparal liquid, using an Olympus SZX7 stereomicroscope. Alternatively, dissection was done in alcohol, with subsequent mounting on slides with Canada balsam and using a stereomicroscope MSP 2 and examination with a Leica DM 1000 microscope.

DNA was extracted from some specimens using non-destructive methods allowing subsequent morphological analysis (see Vuataz et al. 2011 for details). We amplified a 658-bp fragment of the mitochondrial gene cytochrome oxidase subunit 1 (COI) using the primers LCO 1490 and HCO 2198 (Folmer et al. 1994). Sequencing was done with Sanger’s method (Sanger et al. 1977). The genetic variability between specimens was estimated using Kimura 2-parameter distances (K2P; Kimura 1980), calculated with the program MEGA 11 (Tamura et al. 2021; http://www.megasoftware.net).

The GenBank accession numbers are given in the section of type material.

Larvae in toto were photographed using a Canon EOS 6D camera and processed with Adobe Photoshop Lightroom (http://www.adobe.com) and Helicon Focus v. 5.3 (http://www.heliconsoft.com). Pictures of larval and subimaginal parts were taken with a DMC 4500 camera on a Leica M205C stereomicroscope or an Olympus SC 50 camera on an Olympus BX43 microscope and processed with Olympus cellSens v. 3.2. Photographs were subsequently enhanced with Adobe Photoshop Elements 13.

The distribution map was generated with the program SimpleMappr (https://simplemappr.net; Shorthouse 2010).

The terminology follows Kluge (2004). The term "microlepides" is used according to Kluge (2022).

### Abbreviations

**MZL** Muséum cantonal des Sciences Naturelles, Lausanne (Switzerland)

**SPbU** Saint-Petersburg State University (Russia)

## Results

### Siphliops gen. nov.

Taxon classificationAnimaliaEphemeropteraBaetidae

7FC4271D-288F-58F6-B973-F5B45372CCE3

https://zoobank.org/831F7465-0011-4966-8DCF-F28BA399474E

Figs 1, 2, 3, 4, 5, 6, 7, 8

#### Type species.

*Siphliops extenso* gen. nov. et sp. nov., by present designation.

**Figure 1.**
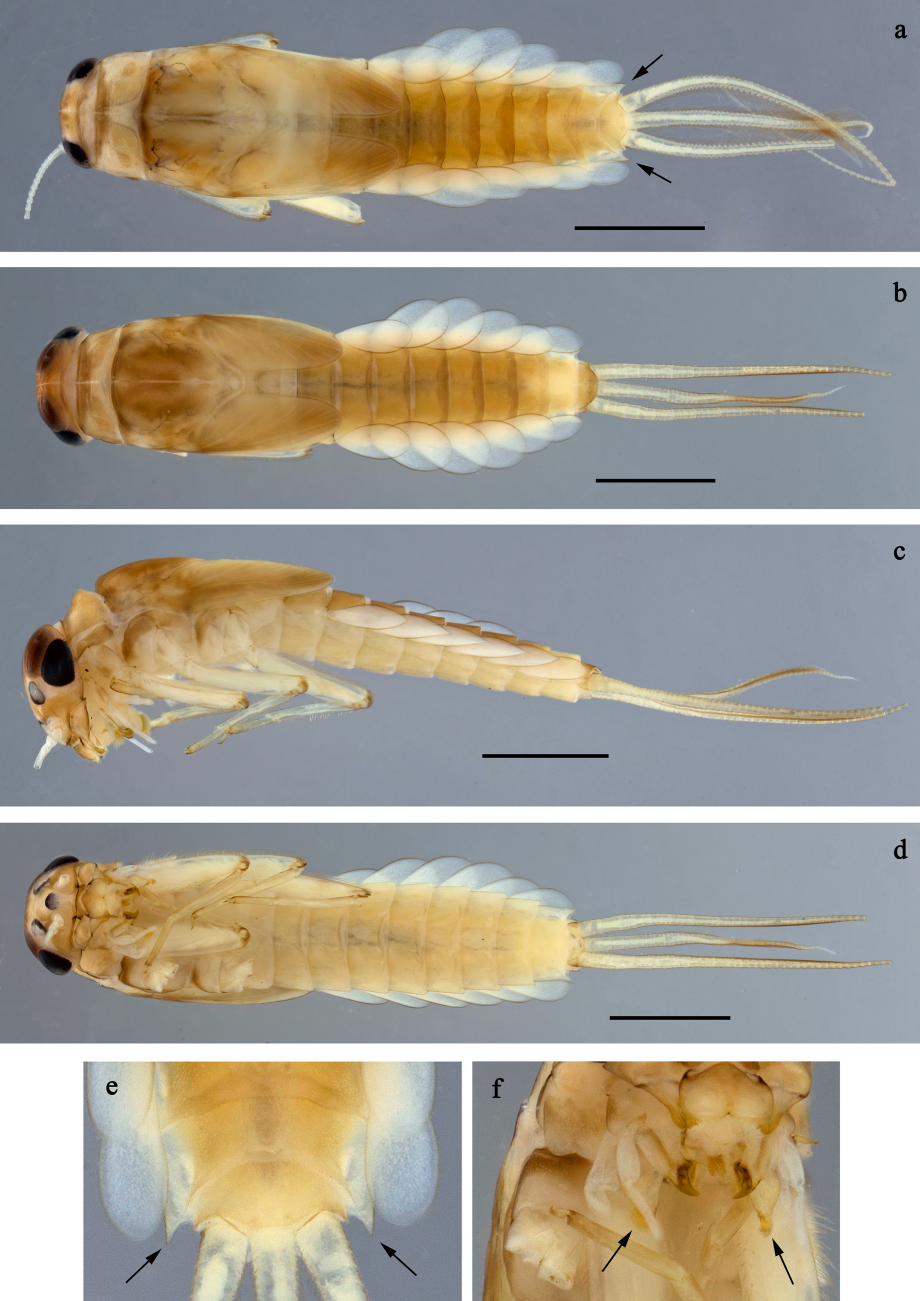
*
Siphliops extenso* gen. nov. et sp. nov., larva, habitus. **a**. Female, dorsal view (arrows: posterolateral extension of tergum IX); **b**. Male, dorsal view; **c**. Male, lateral view; **d**. Male, ventral view; **e**. Abdominal terga VIII–X (arrows: posterolateral extension of tergum IX) **f** mouthparts (arrows: paraglossae). Scale bars: 1 mm.

#### Diagnosis.

**Figure 2.**
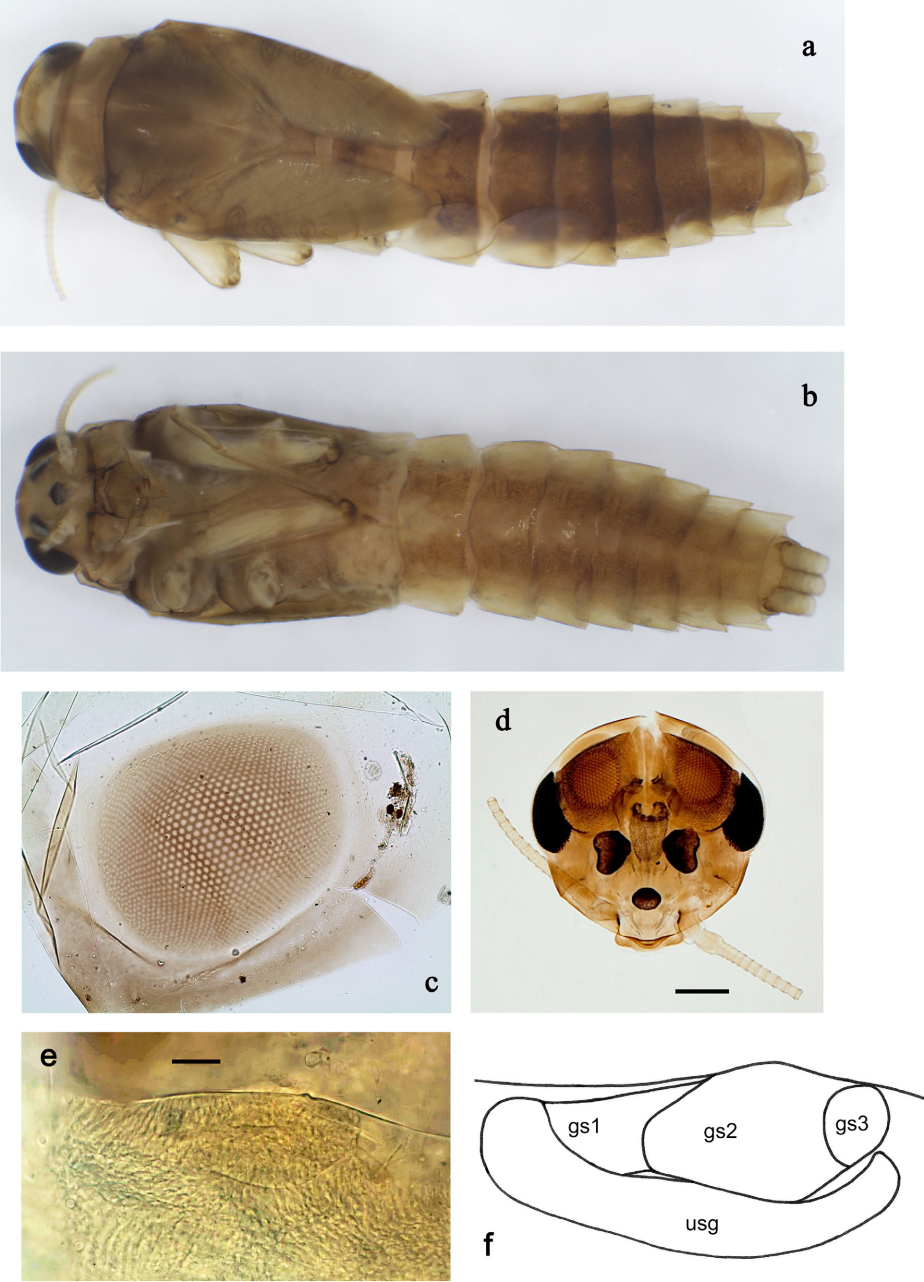
*
Siphliops extenso* gen. nov. et sp. nov., larva. **a**. Dorsal view, hypodermic colour; **b**. Ventral view, hypodermic colour. **c**. Male, developing turban eye; **d**. Male head; **e, f**. Subimaginal gonostylus developing under larval cuticle (premature male larva; usg: unistyliger, gs1–gs3: segments of gonostylus). Scale bars: 100 µm (**d**); 10 µm (**e**).

**Figure 3.**
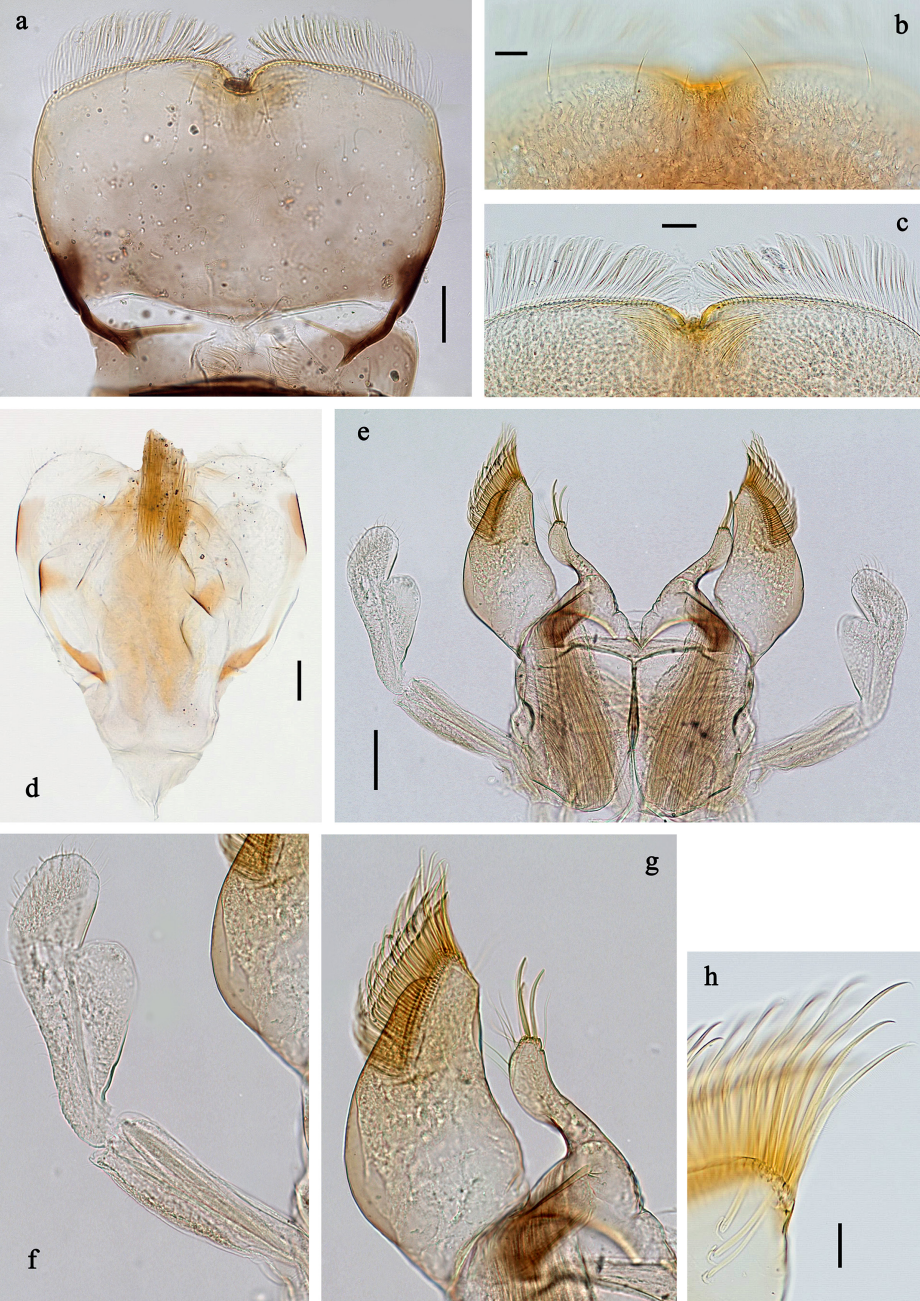
*
Siphliops extenso* gen. nov. et sp. nov., larva. **a**. Labrum; **b**. Labrum, distal section, dorsal focus; **c**. Labrum, distal section, ventral focus; **d**. Hypopharynx and superlinguae; **e**. Labium; **f**. Labial palp; **g**. Glossa, paraglossa; **h**. Paraglossa, apical section. Scale bars: 50 µm (**e**); 20 µm (**a, d**); 10 µm (**b, c, h**).

**Figure 4.**
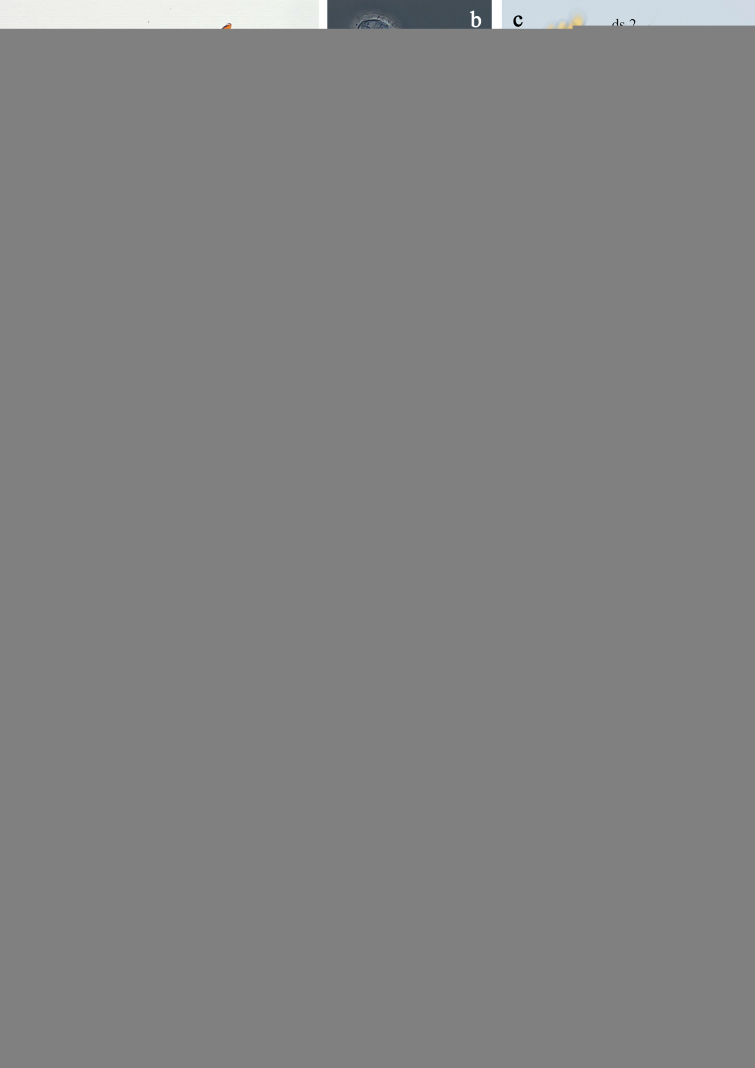
*
Siphliops extenso* gen. nov. et sp. nov., larva. **a**. Maxilla; **b**. Maxillary palp; **c**. maxilla, apical section (ds 1, 2, 3: denti-seta 1, 2, 3); **d**. Right mandible; **e**. Left mandible; **f**. Caudalii; **g**. Paracercus, section; **h**. Cercus, section. Scale bars: 100 µm (**f**); 50 µm (**a**); 20 µm (**d, e**); 10 µm (**b, c, g, h**).

**Figure 5.**
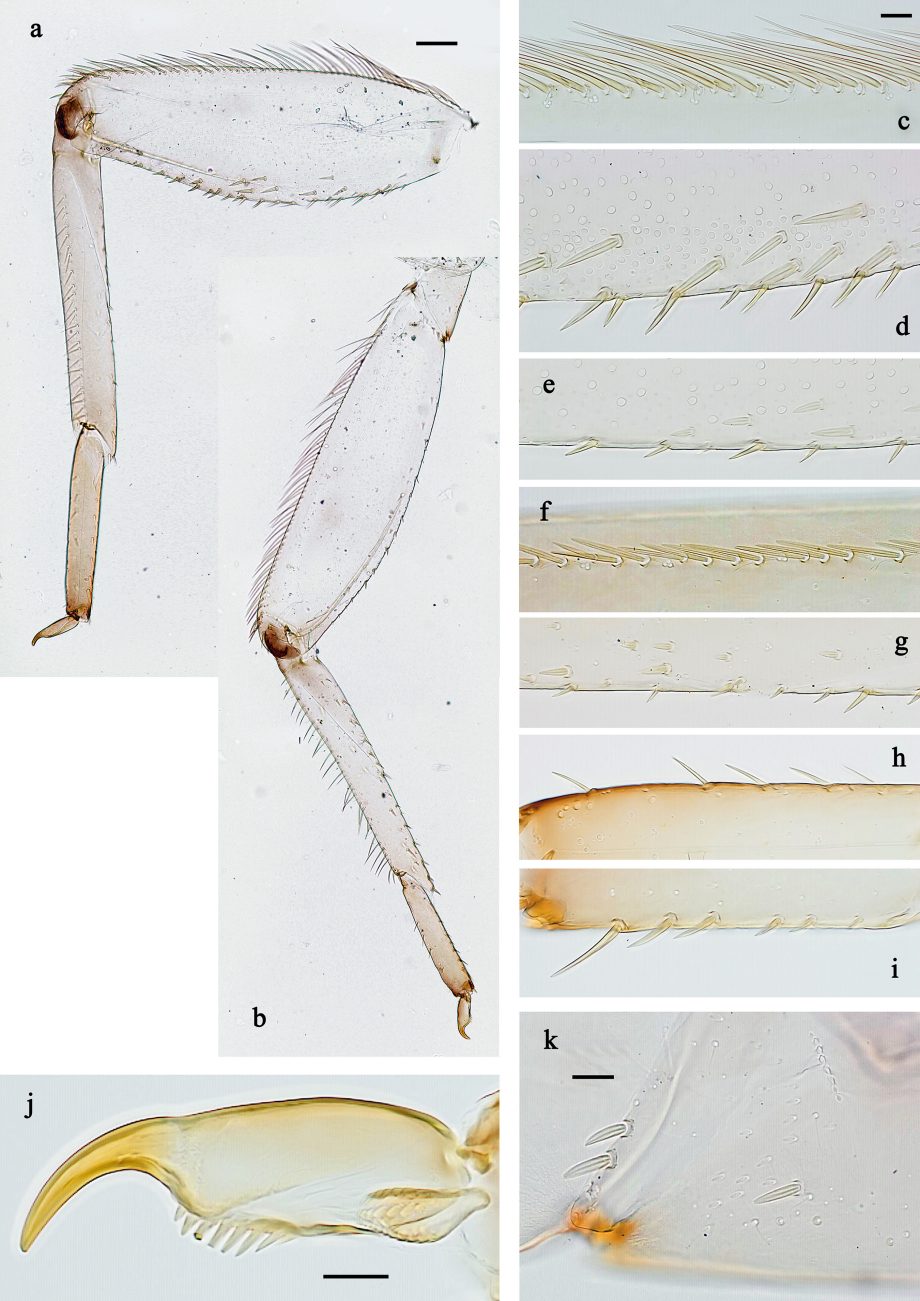
*
Siphliops extenso* gen. nov. et sp. nov., larva. **a**. Fore leg; **b**. Hind leg; **c**. Fore femur, outer margin; **d**. Fore femur, inner margin; **e**. Middle femur, inner margin; **f**. Fore tibia, anterior side; **g**. Fore tibia, inner margin; **h**. Hind tarsus, outer margin; **i**. Hind tarsus, inner margin; **j**. Fore claw; **k**. Middle coxa. Scale bars: 50 µm (**a**); 10 µm (**c–k**).

**Figure 6.**
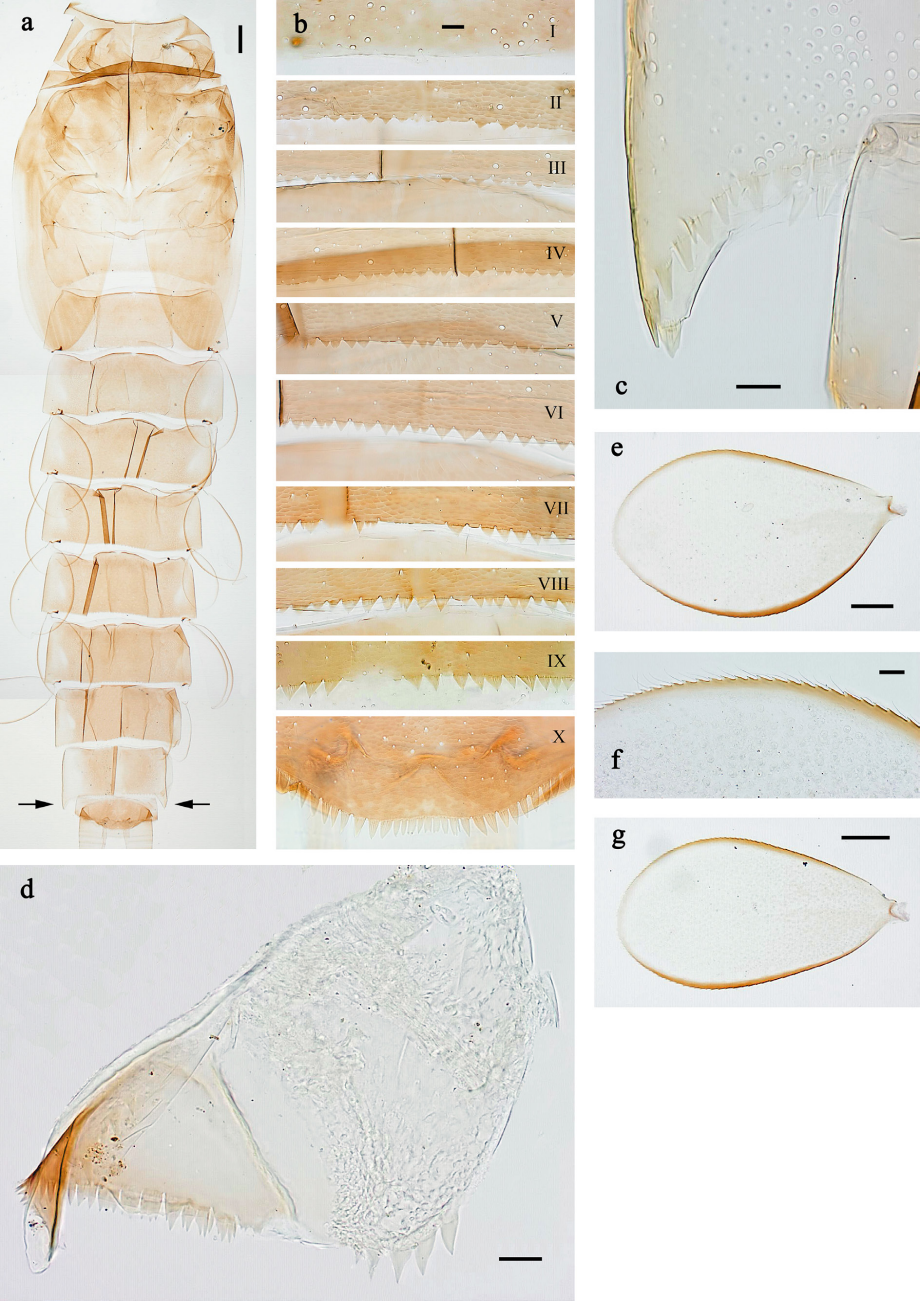
*
Siphliops extenso* gen. nov. et sp. nov., larva. **a**. Abdomen (arrows: posterolateral extension of tergum IX); **b**. Abdominal terga; **c**. Posterolateral extension of abdominal tergum IX; **d**. Paraproct; **e**. Tergalius IV; **f**. Tergalius IV, margin; **g**. Tergalius VII. Scale bars: 100 µm (**a**); 50 µm (**e, g**); 10 µm (**b–d, f**).

***Larva***. The following combination of characters distinguishes *Siphliops* gen. nov. from all other genera of Baetidae: frons with carina-like elevation (Fig. 2d); both mandibles with incisors and kinetodontium fused up to apex, without setae between prostheca and mola (Fig. 4d, e); labium with glossae and paraglossae spread, glossae much shorter than paraglossae (Fig. 3e); labial palp segment II with distolateral protuberance (Fig. 3f); femur with dense row of long, pointed setae at outer margin (Fig. 5a–c); tibia with dense row of long, robust, pointed setae on outer, anterior side (Fig. 4a, b, f); claw markedly broadened in basal 2/3, tapering finger-like in distal 1/3, with single row of denticles (Fig. 5j); posterolateral corners of abdominal tergum IX with acute prolongation (Figs 1a, e, 6a, c); cerci and paracercus well developed, with dense, long swimming setae, in cerci limited to basal half (Fig. 4f); subimaginal gonostyli developing under cuticle of male last instar larva folded in “*Labiobaetis*-type” (Fig. 2e, f; Kluge 2004: 99).

#### Description.

***Larva*** (Figs 1, 2, 3, 4, 5, 6).

***Head*** (Fig. 2d). Frons with carina-like elevation between base of antennae.

***Labrum*** (Fig. 3a–c). Subrectangular, much wider than long. Distal margin with medial emargination and small process. Dorsally with pair of long, simple, sub-median setae, and few long, simple, submarginal setae.

***Right mandible*** (Fig. 4d). Incisor and kinetodontium fused till apex; incisor and kinetodontium with apically rounded denticles; inner margin of innermost denticle of kinetodontium with row of thin setae; prostheca stick-like, distally widened, and denticulate.

***Left mandible*** (Fig. 4e). Incisor and kinetodontium fused till apex; incisor and kinetodontium with apically rounded denticles; prostheca robust, distally denticulate and with short, comb-like structure.

***Hypopharynx*** (Fig. 3d). Apex of lingua with well-developed bunch of spine-like setae.

***Maxilla*** (Fig. 4a–c). Apically with three slender, pointed canines and three denti-setae; distal denti-seta tooth-like, bent in same direction as canines; other denti-setae far separated, slender, bifid, and pectinate; maxillary palp with two segments, apex rounded.

***Labium*** (Fig. 3e–h). Glossae and paraglossae spread. Glossae much shorter than paraglossae, basally broad, narrowing toward middle, distal half finger-like; inner margin with one long, spine-like seta in apical area; outer margin with row of long, spine-like setae in apical area; on apex with three long, spine-like, pectinate setae; ventral surface with numerous medium to long fine, simple scattered setae. Paraglossae basally and medially wide, narrowing distally; inner margin slightly concave; outer margin strongly convex; apex with three rows of long, robust, distally pectinate setae; dorsally with row of long, spine-like setae near inner margin. Labial palp with three segments, segment II with well-developed, distomedial thumb-like protuberance.

***Thorax*. *Hind protoptera*** absent.

***Legs*** (Fig. 5a–k). ***Coxa*** with few spine-like setae, similar to setae along inner margin of femur. ***Femur***. Outer margin with dense row of long, spine-like, pointed setae; with stout, pointed setae along inner margin; femoral patch absent on all legs. ***Tibia*** with dense row of long, robust, pointed setae on outer, anterior side; with short to medium, spine-like setae on and along inner margin. ***Tarsus***. Outer margin with row of medium, spine-like setae; inner margin with row of short to medium, spine-like setae, increasing in length distally, distalmost seta much longer than other setae. ***Claw*** broadened in basal 2/3, finger-like in distal 1/3, with single row of denticles.

***Abdomen*. *Terga*** (Fig. 6a–c). Posterior margins with triangular, pointed spines. Tergum IX distolaterally with acute prolongation.

***Tergalii*** (Fig. 6e–g). Consisting of one lamella; present on segments II–VII.

***Caudalii*** (Fig. 3f). Paracercus well developed; paracercus and cerci with dense, long swimming setae, in cerci limited to basal half.

***Paraproct*** (Fig. 6d). Posterior margin with stout spines, not produced apically. Cercotractor with numerous, small, marginal spines.

***Larval protogonostyli*** (Fig. 2e, f). Based on the investigation of one premature male larva, subimaginal gonostyli developing under cuticle are folded in “*Labiobaetis*-type”: segments II and III directed medially and slightly bent, most caudal point belongs to segment II.

**Figure 7.**
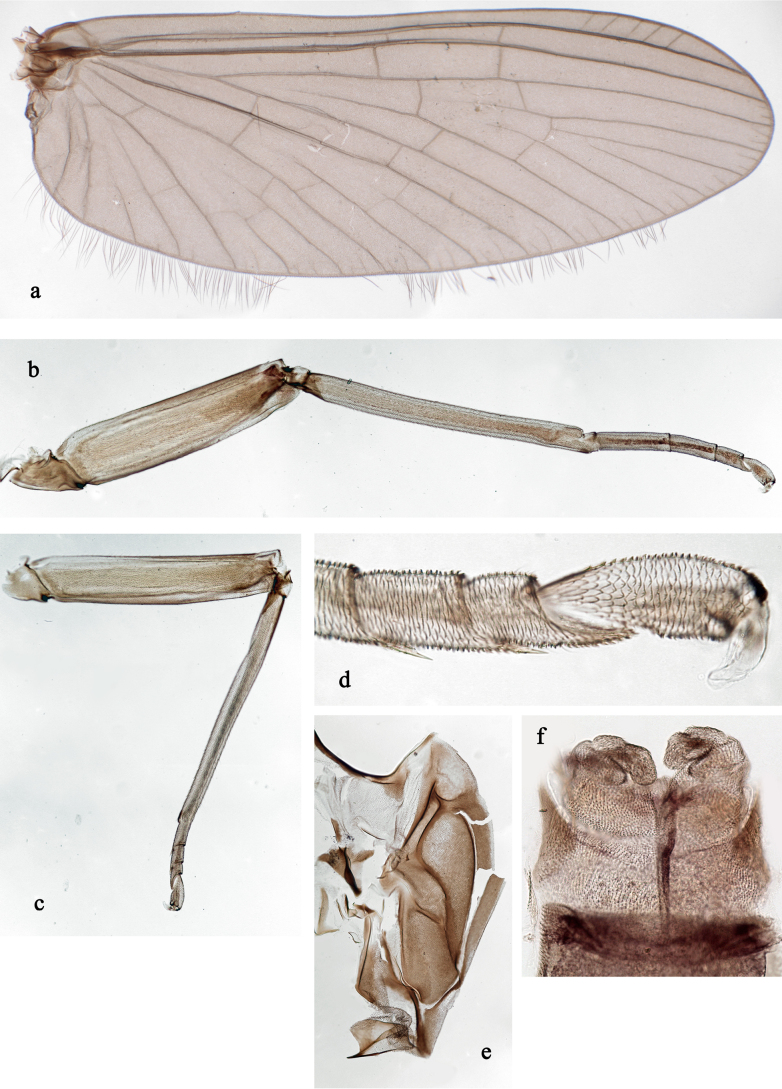
*
Siphliops extenso* gen. nov. et sp. nov., male subimago. **a**. Fore wing; **b**. Fore leg; **c**. Hind leg; **d**. Hind tarsus; **e**. Mesonotum; **f**. Genitalia.

**Male subimago** (Fig. 7).

***Thorax***. Forewings with double intercalary veins; tarsal segments of all legs of both sexes covered with blunt microlepides (Fig. 7d). ***Hind wings*** absent.

#### Etymology.

*Siphliops* is an arbitrary combination of *Siphl*-, with reference to the genus *Siphlonurus* Eaton, 1868 (Siphlonuridae), which has a similar abdomen with posterolateral extensions, and the Greek -*iops* (minnow), with reference to Baetidae, which look and move like small fishes. Gender is masculine.

#### Distribution

**Figure 8.**
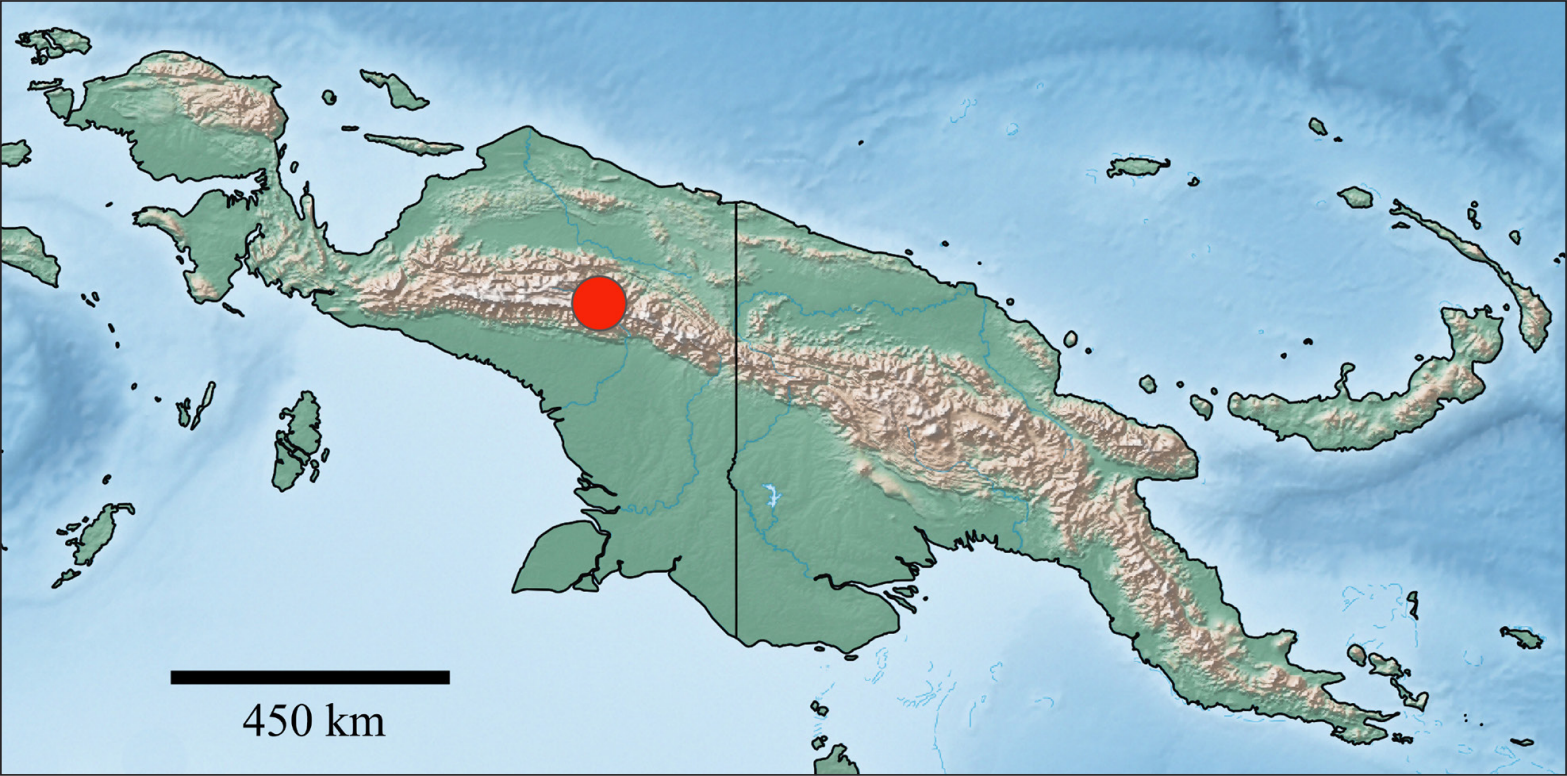
*
Siphliops extenso* gen. nov. et sp. nov., distribution.

**(Fig. 8)**. New Guinea.

### Siphliops extenso

Taxon classificationAnimaliaEphemeropteraBaetidae

gen. nov. et sp. nov.

D684A970-8448-59BE-9F43-1E4A14427BB0

https://zoobank.org/3EF65073-325C-49C1-AB5D-2566FB596FE6

Figs 1, 2, 3, 4, 5, 6, 7, 8

#### Type material.

***Holotype***. Indonesia • female larva; Highland Papua Prov., Wamena; 04°06'38"S, 138°55'49"E; 1650 m; 27. vii. 2015; leg. S. Niderer; on slide; GBIFCH01221843; MZL. ***Paratypes***. 23 larvae; same data as holotype; 6 on slides; GBIFCH01221829, GBIFCH01221844, GBIFCH01221845 (protogonostyli); GBIFCH01221846 (abdominal sterna), GBIFCH01588927 (GenBank PX697239), GBIFCH01588928 (GenBank PX697240); MZL; 17 in alcohol; GBIFCH01581945, GBIFCH01581946, GBIFCH01582033, GBIFCH01582034, GBIFCH01582066, GBIFCH01588929 (GenBank PX697241); MZL • 1 L-S♂ (on slide), 1 L/S♀(on slide), 4 larvae (in alcohol); Highland Papua Prov., Baliem valley, Wamena, river Elagaima, 15–19. viii.2012, leg. N. Kluge & L. Sheyko; SPbU.

#### Description.

***Larva*** (Figs 1, 2, 3, 4, 5, 6). Body length 4.5–5.8 mm. Cerci ~0.9 × body length, paracercus ~0.5 × body length.

***Cuticular colouration*** (Fig. 1a–d). Head, thorax and abdomen dorsally brown, mesonotum darker, pronotum darker along anterior margin. Head, thorax and abdomen ventrally light brown. Legs light brown, apex of femur, tarsus, and claw darker. Caudalii light brown.

***Hypodermic colouration***. Dorsally and ventrally dark brown as in Fig. 2a, b.

***Head*** (Fig. 2d). Frons with carina-like elevation.

***Antenna***. Length ~1.5 × head length.

***Labrum*** (Fig. 3a–c). Subrectangular, length ~0.6 × maximum width. Distal margin almost straight, medially with nearly V-shaped emargination and small process. Dorsally with pair of sub-median setae and two long, simple, submarginal setae on each side; fine, simple setae scattered on surface. Ventral margin with dense row of long, flattened, apically slightly bent setae.

***Right mandible*** (Fig. 4d). Incisor with four denticles, outermost denticle shorter and turned ventrally; kinetodontium with five denticles, innermost denticle with row of fine, simple setae on lateral margin. Prostheca stick-like, apically widened, and denticulate, leaned against kinetodontium. Margin between prostheca and mola almost straight, with row of minute denticles. Tuft of setae on proximal corner of mola present.

***Left mandible*** (Fig. 4e). Incisor with five denticles, outermost denticle shorter and turned ventrally; kinetodontium with three denticles. Prostheca robust stick-like, apically widened, denticulate and with short, comb-like structure. Margin between prostheca and mola slightly concave. Tuft of setae on proximal corner of mola absent.

***Hypopharynx and superlinguae*** (Fig. 3d). Lingua shorter than superlinguae, apically with well-developed, broad bunch of spine-like setae. Superlinguae distally rounded; lateral margins rounded; fine, long, simple setae along distal margin.

***Maxilla*** (Fig. 4a–c). Galea-lacinia ventrally with two simple setae just proximad of canines. Medially with one pectinate, spine-like seta, and row of four simple setae. Maxillary palp longer than galea-lacinia; palp segment II subequal in length to segment I, conspicuously thinner than segment I, apex rounded; setae on maxillary palp fine, simple, scattered over surface of segments I and II.

***Labium*** (Fig. 3e–h). Glossae bent toward middle, inner margin with one long, spine-like seta in apical area; apex with three long, robust, spine-like, pectinate setae; outer margin with six or seven long, spine-like setae in apical area; paraglossae wide, narrowing distally; inner margin slightly concave; outer margin strongly convex; apex with three rows of long, robust, distally pectinate setae, proceeding on ventral surface; dorsally with row of three long, spine-like setae near inner margin. Labial palp with segment I 0.7 × length of segments II and III combined. Segment II with well-developed, thumb-like, rounded, distolateral protuberance, dorsally with one spine-like seta near distal outer margin. Segment III oblong, outer margin convex; length ~1.4 × maximal width; at base ~0.6 × as wide as maximal width of segment II; ventral surface with short, spine-like, simple setae and short, fine, simple setae.

***Thorax*. *Hind protoptera*** absent.

***Legs*** (Fig. 5a–k). Ratio of leg segments (femur, tibia, tarsus, claw): fore leg 1.2:1.0:0.4:0.2, middle leg 1.1:1.0:0.4:0.1, and hind leg 1.2:1.0:0.4:0.2. ***Coxa*** with few spine-like setae, similar to setae along inner margin of femur. ***Femur***. Length of fore femur ~3 × maximum width, middle and hind femur slenderer. Outer margin with dense row of long, spine-like, pointed setae; with stout, pointed setae along inner margin (more and larger setae on fore leg, fewer and smaller on middle and hind legs); femoral patch absent on all legs. ***Tibia*** with dense row of long, robust, pointed setae on outer, anterior side; with short to medium-length, spine-like setae on and along inner margin. Patella-tibial suture present on all legs, on basal half. ***Tarsus***. Outer margin with row of medium, spine-like setae; inner margin with row of short to medium-length, curved, spine-like setae, increasing in length distally, distalmost seta much longer than other setae. ***Claw*** much broader in basal 2/3, finger-like in distal 1/3, with single row of seven or eight denticles.

***Abdomen*. *Terga*** (Fig. 6a–c). Surface without scale bases; with scattered micropores, especially in lateral parts. Posterior margin of terga: I smooth, without spines; II–X with triangular, pointed spines, narrower and longer toward X; row of spines on tergum IX interrupted in middle part behind bases of submedian setae. Posterolateral corners of abdominal tergum IX with acute prolongation.

***Sterna***. Posterior margins of sterna smooth, without spines.

***Tergalii*** (Fig. 6e–g). Present on abdominal segments II–VII; tergalii III–V oblique oval, II, VI and VII oblong. Tracheation not visible. Margin with small denticles intercalating short, fine, simple setae; costal and anal margins brown.

***Paraproct*** (Fig. 6d). Posterior margin with ~5 stout spines. Cercotractor with numerous, marginal, apically split spines.

***Caudalii*** (Fig. 4f–h). Cerci and paracercus with densely packed, long swimming setae; distal half of cerci without swimming setae.

***Pose of subimaginal gonostyli under cuticle*** (Fig. 2e, f). Based on one premature male larva, subimaginal gonostyli developing under cuticle folded in “*Labiobaetis*-type”: segments II and III directed medially and slightly bent, most caudal point belongs to segment II.

**Male subimago** (Fig. 7). Fore wing length 5 mm.

***Head*** brown. Antennal scape and pedicel brown, flagellum ochre. Turbinate eyes cylindrical, not widened distally, with round facetted surfaces; in last instar larva area with large facets limited to central part of facetted surface (Fig. 2c). In subimaginal stage, height of turbinate eye less than its width; facetted surface dull reddish brown, stem brownish with ochre, with lighter ring bordering facetted surface.

***Thorax***. Subimaginal cuticle with brown and colourless areas, mesonotum brown (Fig. 7e); imaginal cuticle ochre-brown, equally dark on dorsal, ventral, and lateral surfaces.

***Fore wings*** with subimaginal cuticle evenly brown (Fig. 7a) due to brown rings surrounding base of each microtrichium.

***Hind wings*** absent.

***Legs*** (Fig. 7b–d). Subimaginal cuticle mostly ochre with dark-brown microtrichia; femur with brownish outer side and area near apex; tibia with dark-brown markings near base and on inner-apical corner; tarsomeres with dark-brown distal borders. Hypodermis mostly ochre, with reddish-brown apex of femur. In both sexes, tarsus of middle and hind legs relatively short, with two long apical spines (on initial 2^nd^ and 3^rd^ tarsomeres); fore tarsus of female (examined in larva ready to moult) with two long apical spines on 2^nd^ and 3^rd^ tarsomeres. On all legs of both sexes subimaginal cuticle of last tarsal segment covered with blunt microlepides, each with one or two small points; on other tarsomeres subimaginal cuticle covered with blunt microlepides (Fig. 7d).

***Abdomen***. Subimaginal cuticle mostly ochre with dark-brown microtrichia. Hypodermis with dark-brown hypodermal colouration as in mature larva (as in Fig. 2a, b), all abdominal terga nearly uniformly dark brown with lateral margins lighter ochre; abdominal sterna mostly brown with lateral and median parts lighter ochre.

***Gonostyli***. Judging from the undeveloped genitalia of the examined specimen, distal segments of gonostyli are relatively long (Fig. 7f).

**Imago**. Unknown.

**Egg**. Unknown.

#### Etymology.

The specific epithet refers to the conspicuous posterolateral, acute extensions of abdominal tergum IX.

#### Distribution.

New Guinea (Fig. 8).

#### Genetics.

The three COI sequences obtained from specimens of *Siphliops extenso* gen. nov. et sp. nov. from the same location share the same haplotype (K2P 0%), as it is expected in such a case.

## Discussion

The new genus *Siphliops* gen. nov. obviously belongs to the family Baetidae, based on the turban eyes of the male imago (visible in male larva and subimago; Fig. 2c, d), the fore wing with double intercalary veins (Fig. 7a), and a series of larval characters—e.g. Y-shaped frontal suture ventral of lateral ocelli, labrum with distinctly expressed medial incision (Fig. 3a), kinetodontium fused with mandible and with incisor (Fig. 4d, e), left prostheca stout and stick-like, apically denticulate (Fig. 4e), femur with apical anterior outer projection curved toward inner side of femur (Fig. 5a, b) (Kluge 2004; Kluge and Novikova 2011).

Based on the rank-free system of Kluge (Kluge 2004; Kluge and Novikova 2011), *Siphliops* gen. nov. belongs to the Anteropatellata, because the patella–tibial suture is present on all legs of the larva, including fore legs (Fig. 5a, b); to Baetovectata because of forewings with double intercalary veins (Fig. 7a), and 2^nd^ segment of subimaginal gonostylus developing under larval cuticle bent caudally or medially, but not laterally (Fig. 2e, f); and to Baetungulata or Baetinae (sensu Kazlauskas 1972) because of claws with one row of denticles on the inner anterior side (Fig. 5j) and a maxillary palp with two segments (Fig. 4a) (Kluge and Novikova 2011). Finally, the new genus is part of the “non-Baetofemorata” or the “non-*Baetis* complex” sensu Waltz and McCafferty (1997), because larval legs have no femoral patch (Kluge and Novikova 2011). However, the male subimago of “non-Baetofemorata” usually have the tarsomeres of the middle and hind legs and the 5^th^ tarsomere of the fore leg covered with pointed microlepids (Kluge and Novikova 2011), whereas the subimagos of both sexes of the new genus have blunt microlepides on all tarsomeres of all legs (Fig. 7d).

The new genus *Siphliops* gen. nov. belongs to the tribus Labiobaetini Kluge & Novikova, 2016 according to the diagnosis of this tribus by Kluge and Vishnevskaya (2026): in male genitalia, the styliger muscle is completely absent; the subimaginal gonostyli developing under the larval cuticle are medially directed (either of the “*Labiobaetis*-type” or of the “*Acentrella*-type”) (Fig. 2e, f); in the larval labium, paraglossae are more or less strengthened and widened, with the exception of strongly modified forms (Fig. 3e, g). Compared to other genera of Labiobaetini, *Siphliops* gen. nov. has a similar setation on the femur and tibia as *Papuanatula*, but also many strong differences (e.g. in labrum, labium, mandibles, caudalii; for details see Kaltenbach 2025: table 3, fig. 5; Kaltenbach et al. 2025a); the labial palp is similar to *Labiobaetis*, but amongst other characters, leg setation is very different, and also labrum setation and labium (see Kaltenbach and Gattolliat 2018; Kaltenbach et al. 2025b); the shape of the claw is similar to *Pseudopannota camerunense* (Ulmer, 1920), but many other characters are very different (e.g. in labrum, labium, maxilla, legs; see Kluge and Novikova 2016).

As a conclusion, apart from the diagnostic characters of Labiobaetini, there are only few morphological affinities between *Siphliops* gen. nov. and other genera of Labiobaetini, which is generally in line with affinities between most genera of Labiobaetini.

Remarkably, larvae of *Siphliops* gen. nov. were collected independently by two research teams during separate field expeditions. Given the short distance between the two sampling sites, these specimens clearly represent a single population. Despite extensive sampling across New Guinea, encompassing many sites, *Siphliops* gen. nov. has not been recorded from any other region of the island. The type locality and the other known site do not appear to exhibit distinctive habitat characteristics, nor does the new genus show evidence of adaptation to a specialized ecological niche. It is therefore surprising that no additional populations have been reported elsewhere in New Guinea. However, considering the size and the highly structured topography of the island and its limited accessibility, with many regions remaining poorly explored, the discovery of additional populations or even new species can be expected in the future.

## Supplementary Material

XML Treatment for Siphliops

XML Treatment for Siphliops extenso

## Additional information

### Use of AI

No use of AI was reported.

### Author ORCIDs

Thomas Kaltenbach https://orcid.org/0000-0001-8052-0388

Nikita J. Kluge https://orcid.org/0000-0001-9741-7790

Martin Bláha https://orcid.org/0000-0003-3597-6604

Jiří Patoka https://orcid.org/0000-0002-2797-0563

Jean-Luc Gattolliat https://orcid.org/0000-0001-5873-5083

### Data availability

All of the data that support the findings of this study are available in the main text
